# Effect of 1-Carbaldehyde-3,4-dimethoxyxanthone on Prostate and HPV-18 Positive Cervical Cancer Cell Lines and on Human THP-1 Macrophages

**DOI:** 10.3390/molecules26123721

**Published:** 2021-06-18

**Authors:** Rui Medeiros, Bruno Horta, Joana Freitas-Silva, Jani Silva, Francisca Dias, Emília Sousa, Madalena Pinto, Fátima Cerqueira

**Affiliations:** 1 Molecular Oncology and Viral Pathology GRP—IC, Portuguese Institute of Oncology of Porto (IPO Porto), Rua Dr. António Bernardino de Almeida, 4200-072 Porto, Portugal; bhorta@porto.ucp.pt (B.H.); jani.silva@aquavalor.pt (J.S.); francisca.carvalho.dias@ipoporto.min-saude.pt (F.D.); fatimaf@ufp.edu.pt (F.C.); 2 FP-ENAS Research Unit, UFP Energy, Environment and Health Research Unit, CEBIMED, Biomedical Research Centre, Fernando Pessoa University, Praça 9 de Abril, 349, 4249-004 Porto, Portugal; 3 Faculty of Health Sciences, Fernando Pessoa University, Rua Carlos da Maia, 296, 4200-150 Porto, Portugal; 4 ICBAS—Instituto de Ciências Biomédicas Abel Salazar, Universidade do Porto, Rua de Jorge Viterbo Ferreira, 228, 4050-313 Porto, Portugal; 5 Associated Laboratory, CBQF—Centre for Biotechnology and Fine Chemistry, Higher School of Biotechnology, Portuguese Catholic University, Rua Diogo Botelho, 1327, 4169-005 Porto, Portugal; 6 CIIMAR/CIMAR, Interdisciplinary Centre of Marine and Environmental Research, Terminal de Cruzeiros do Porto de Leixões, Av. General Norton de Matos s/n, 4450-208 Matosinhos, Portugal; joanafreitasdasilva@gmail.com (J.F.-S.); esousa@ff.up.pt (E.S.); madalena@ff.up.pt (M.P.); 7 AquaValor—Centro de Valorização e Transferência de Tecnologia da Água, Rua Dr. Júlio Martins, nº1, 5400-342 Chaves, Portugal; 8 Laboratory of Organic and Pharmaceutical Chemistry, Department of Chemical Sciences, Faculty of Pharmacy, University of Porto, Rua Jorge Viterbo Ferreira, 228, 4050-313 Porto, Portugal

**Keywords:** 1-carbaldehyde-3,4-dimethoxyxanthone, antitumor, prostate cancer, cervical cancer, immunomodulation, macrophage functions, cytokines

## Abstract

Xanthone derivatives have shown promising antitumor properties, and 1-carbaldehyde-3,4-dimethoxyxanthone (**1**) has recently emerged as a potent tumor cell growth inhibitor. In this study, its effect was evaluated (MTT viability assay) against a new panel of cancer cells, namely cervical cancer (HeLa), androgen-sensitive (LNCaP) and androgen-independent (PC-3) prostate cancer, and nonsolid tumor derived cancer (Jurkat) cell lines. The effect of xanthone **1** on macrophage functions was also evaluated. The effect of xanthone **1**-conditioned THP-1 human macrophage supernatants on the metabolic viability of cervical and prostate cancer cell lines was determined along with its interference with cytokine expression characteristic of M1 profile (IL-1 ≤ β; TNF-α) or M2 profile (IL-10; TGF-β) (PCR and ELISA). Nitric oxide (NO) production by murine RAW264.7 macrophages was quantified by Griess reaction. Xanthone **1** (20 μM) strongly inhibited the metabolic activity of the cell lines and was significantly more active against prostate cell lines compared to HeLa (*p* < 0.05). Jurkat was the cell most sensitive to the effect of xanthone **1**. Compound **1**-conditioned IL-4-stimulated THP-1 macrophage supernatants significantly (*p* < 0.05) inhibited the metabolic activity of HeLa, LNCaP, and PC-3. Xanthone **1** did not significantly affect the expression of cytokines by THP-1 macrophages. The inhibiting effect of compound **1** observed on the production of NO by RAW 264.7 macrophages was moderate. In conclusion, 1-carbaldehyde-3,4-dimethoxyxanthone (**1**) decreases the metabolic activity of cancer cells and seems to be able to modulate macrophage functions.

## 1. Introduction

Xanthones, specifically methoxyxanthones, have already been described by our group as possessing antitumor and immunomodulatory activities [1,2,3,4]. Methoxyxanthone derivatives have been shown to inhibit the growth of human cancer cell lines (breast, melanoma, and renal) [2] as well as the proliferation of human peripheral blood mononuclear cells [2] and nitric oxide (NO) production by J774 macrophages [1].

**Figure 1:**
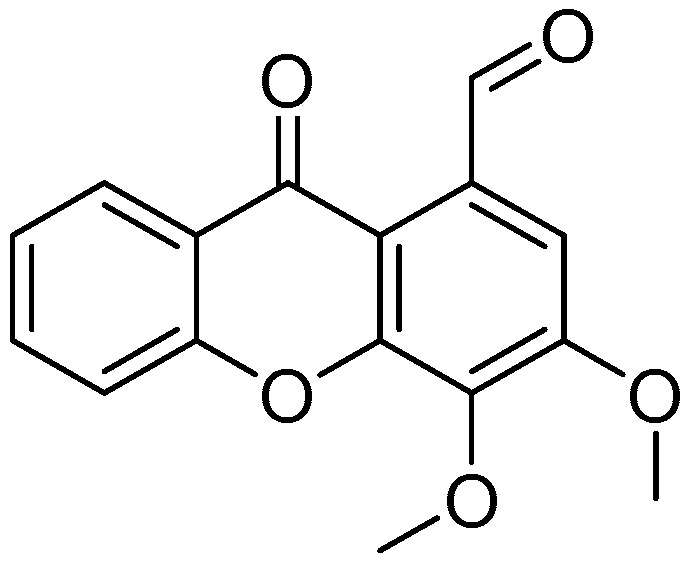
Chemical structure of 1-carbaldehyde-3,4-dimethoxyxanthone (**1**).

1-carbaldehyde-3,4-dimethoxyxanthone (**1**) (Figure 1) was previously synthesized by our group, and its antitumor effect was evaluated using cancer cell lines of different origins [5,6]. The study of xanthone **1** on neuroblastoma revealed that this compound is a TAp73 activator [6].

Nowadays, the fight against cancer entails the development of new therapeutic approaches, such as cancer immunotherapy.

The tumor microenvironment (TME) is important in all stages of tumor development, and the interaction between the tumor and the cells around it defines the final outcome of the disease [7,8]. Tumor-associated macrophages (TAMs), the more representative type of cell in TME, are seen as potentially useful targets for cancer immunotherapy [7,9] Macrophages can adopt a phenotype that changes between classically activated (M1) to alternatively activated (M2), with M1 exerting a proinflammatory and antitumor function, while M2 is mainly beneficial for tumors because of its tumor-promoting, angiogenic, and immunosuppressive effects [7,9]. Polarization into M1 can be achieved by lipopolysaccharide (LPS) stimulation, and macrophages will produce cytokines such as TNF-α, IL-1, and IL-6 [9]. The M2a phenotype is induced when TAMs are stimulated with IL-4 and/or IL-13, and they will produce high amounts of IL-10 and TGF-β that exert tumor-promoting activities [9,10].

From some previous studies, it can be inferred that an increase in the number of TAMs is associated with a poor outcome. However, the M1/M2 ratio is also noted as being crucial for the prognosis of disease [11,12,13]. The number of TAMs and the proportion of M1 and M2 cells have been found to be associated with tumor progression and a worse prognosis for solid tumors, such as cervical or prostate [11,12,14]. Therapy strategies targeting TAMs include decreasing macrophage density, either by cytotoxicity or inhibition of recruitment, and redirecting TAMs to an M1 phenotype [9,15,16].

In this work, the antitumor activity of xanthone **1** was tested against liquid and solid tumors using lymphocyte (Jurkat), cervical (HeLa), and androgen-sensitive (LNCaP) and androgen-independent (PC-3) prostate cancer cell lines. The effect of culture supernatants from unstimulated, LPS-stimulated, or IL-4-stimulated macrophages treated with compound **1** on the metabolic viability of solid-derived tumor cell lines was also investigated. To study the interference of xanthone **1** on macrophage function, the expression of cytokine characteristic of M1 (IL-1β and TNF-α) and M2 (TGF-β1 and IL-10) phenotypes by THP-1 human macrophages was evaluated. The effect of xanthone **1** on NO production by LPS-stimulated RAW 264.7 murine macrophage cell line was also tested. This is the first report concerning the effect of xanthone **1** on macrophage function.

## 2. Results

### 2.1. Xanthone ***1*** Decreased the Metabolic Viability of Adherent and Nonadherent Cancer Cell Lines

The effect of xanthone **1** on the metabolic viability of adherent and nonadherent cancer cell lines was evaluated (Table 1).

Compound **1** strongly reduced the metabolic viability of Jurkat and the prostate cell lines tested, but its cytotoxic effect was not so evident against the cervical cancer cell line HeLa.

Considering the effect of the referred xanthone **1** against solid tumor cancer cell lines, at 20 µM, no significant differences were seen for the two prostate cancer cell lines used. However, the cytotoxic effect of xanthone **1** against both LNCaP and PC-3 prostate cancer cell lines was significantly higher compared to the cervical cancer cell line HeLa (*p* < 0.05 and *p* < 0.01, respectively). No significant differences in cytotoxic effect were seen on the different solid tumor cell lines when xanthone **1** was tested at 10 μM. For concentrations of 5 µM, it was observed that the compound was significantly more toxic to the androgen-independent PC-3 prostate cell line compared to the androgen-sensitive prostate cell line LNCaP (*p* < 0.05) or the cervical cancer cell line HeLa (*p* < 0.01).

Analyzing the activity of compound **1** (20 μM) against solid and liquid tumors, it was more active against the nonadherent Jurkat cell line compared to LNCaP (*p* < 0.05) or HeLa (*p* < 0.001). At 10 μM, there was always a significant difference between the effect on Jurkat compared to the other cell lines tested (*p* < 0.01 for PC-3 and *p* < 0.001 for LNCaP and HeLa). However, when the xanthone **1** was tested at 5 µM, significant differences were only observed between the effects on Jurkat versus LNCaP (*p* < 0.05) and Jurkat versus HeLa (*p* < 0.01). Analyzing the results, it seemed that the cytotoxic effect of xanthone **1** was more potent against the Jurkat cell line; however, the differences were not always statistically significant.

### 2.2. Effect of Xanthone ***1*** on Cytokine Expression by THP-1 Macrophages

**Figure 2:**
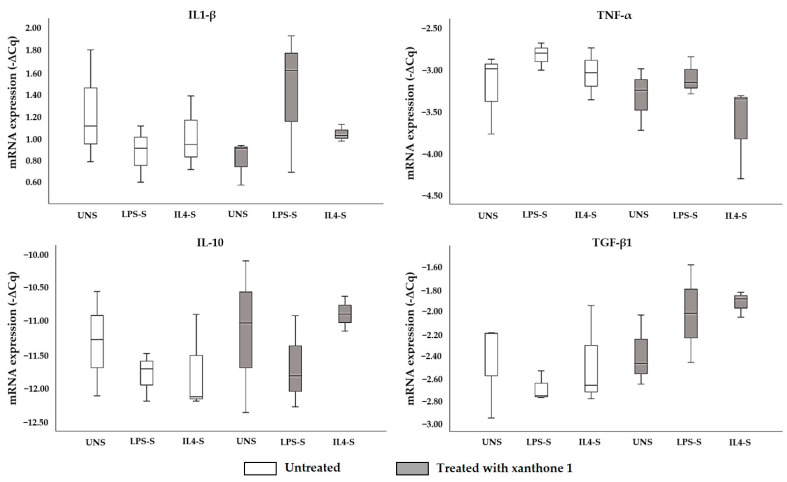
Effect of 1-carbaldehyde-3,4-dimethoxyxanthone (**1**; 10 mM) on the expression of mRNA (−∆Cq) of IL-1β, TNF-α, TGF-β1, and IL-10 cytokines by THP-1 macrophages. UNS: unstimulated; LPS-S: LPS-stimulated; IL4-S: IL4-stimulated. Values represent mean ± SEM (*n* = 3).

The effect of compound **1** on the mRNA expression of cytokines from THP-1 macrophages (unstimulated, LPS-stimulated, or IL-4-stimulated) was evaluated (Figure 2).

Xanthone **1** did not significantly affect the expression of mRNA of the cytokines studied compared to untreated macrophages, irrespective of whether the macrophages were unstimulated or stimulated with LPS or IL-4.

**Figure 3:**
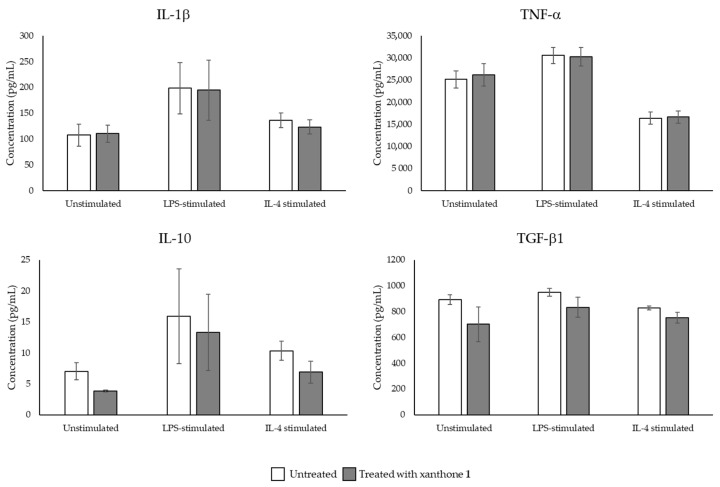
Effect of 1-carbaldehyde-3,4-dimethoxyxanthone (xanthone **1**; 10 μM) on IL-1β, TNF-α, IL-10, and TGF-β1 cytokines produced by THP-1 macrophages. For unstimulated, LPS-stimulated, or IL-4-stimulated macrophages, the level of cytokines was determined after 24 h of incubation for untreated or compound **1**-treated macrophages. Data is expressed as the mean ± SEM (*n* = 3). Cytokine quantification was performed in duplicate.

The effect of xanthone **1** on cytokine expression by the macrophages was also quantified by ELISA (Figure 3).

Independently of the stimulation (unstimulated, LPS-stimulated, or IL-4-stimulated), the treatment of THP-1 macrophages with xanthone **1** did not cause a significant difference in cytokines quantified by ELISA, which is in accordance with the results for mRNA expression.

### 2.3. Xanthone ***1*** Treatment Interferes with Cytotoxic Activity of Macrophage Supernatants against Prostate and Cervical Cancer Cell Lines

**Figure 4:**
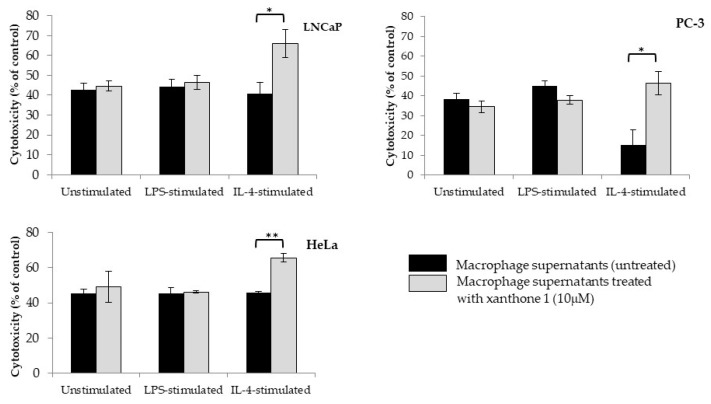
Cytotoxic effect of 1-carbaldehyde-3,4-dimethoxyxanthone (xanthone **1**)-conditioned macrophage supernatants against prostate (LNCaP and PC-3) and cervical (HeLa) cancer cell lines. Unstimulated macrophages and LPS- or IL-4-stimulated macrophages were left untreated or were treated with xanthone **1** (10 μM). The supernatants of the macrophages exposed to the different situations were added to cancer cell lines, and reduction of the metabolic viability was evaluated. LPS, lipopolysaccharide; IL, interleukin. Results show mean ± SEM (*n* = 3–6). * *p* < 0.05 ** *p* < 0.01.

The effect of macrophage supernatants on the metabolic viability of prostate and cervical solid tumor cell lines was evaluated (Figure 4).

The effect of supernatants was evaluated for unstimulated, LPS-stimulated, and IL-4-stimulated macrophages. In all cases, the effect was compared between untreated and xanthone **1**-treated macrophages.

For unstimulated macrophages, no significant differences were observed in the reduction of metabolic activity of the cancer cell lines when comparing xanthone **1**-treated and untreated macrophage supernatants. When macrophages were stimulated with LPS to induce an M1 phenotype, the cytotoxicity of the macrophage supernatants was not significantly affected by compound **1** treatment compared to the untreated ones. When THP-1 macrophages were stimulated with IL-4 in order to induce an M2 phenotype, the treatment with xanthone **1** (10 μM) caused a significant (*p* < 0.05) increase in the toxicity of macrophage supernatants against PC-3, LNCaP, and HeLa cell lines.

### 2.4. Xanthone ***1*** Inhibits the Production of Nitric Oxide by LPS-Stimulated RAW264.7 Macrophages

Xanthone **1** had a moderate inhibitory effect on NO production by LPS-stimulated RAW 264.7 cell line, with no relevant effect on cellular viability (Table 2). Xanthone **1** was able to inhibit NO production by LPS-stimulated RAW 264.7 cell line at concentrations of 10 (28.1 ± 3.7) and 5 (15.3 ± 3.0) μM compared to NO production by the control cell lines.

## 3. Discussion

The antitumor effect of xanthone **1** against neuroblastoma cells has previously been established. Its effect is related to Tap-73 activation, leading to enhanced Tap-73 transcriptional activity, induction of cell cycle arrest, and apoptosis in p53-null and p53-mutant tumor cells [6]. In addition, the absence of Tap-73 is associated with an interference with M1/M2 polarization and the development of chronic inflammation [17].

In the present work, xanthone **1** showed strong antitumor activity against the Jurkat and prostate cell lines, although it was less potent against the HeLa cell line. It is important to note that the poor aqueous solubility and bioavailability of xanthone **1** might have influenced the effects observed for this compound on the viability and metabolic function of the different cell lines [5].

Prostate and cervical cancer are among the most prevalent cancers in men and women, respectively [18,19]. The antitumor effect of xanthone **1** at 20 μM was more evident against the prostate cancer cell line than the cervical cancer cell line, suggesting a selectivity of its antitumor effect, but no differences were observed at 10 μM. Focusing on the prostate cancer cell lines, the effect was significantly different only at the lowest concentration tested (5 μM), and compound **1** was more active against the androgen-independent PC-3 cell line. Regarding the cervical cancer cell line, Oh and coworkers have shown that upregulation of Tap-73 expression regulates paclitaxel-induced apoptosis in HeLa cells, which impairs p53 expression due to the influence of HPV E6 and E7 oncoproteins [20]. Based on our results we can hypothesize that xanthone **1** may not be as powerful as paclitaxel in inducing Tap-73 upregulation in HeLa cells. The Jurkat cell line was used in the study to allow comparison of xanthone **1** activity between cell lines obtained from solid and nonsolid tumors. At 10 μM, xanthone **1** significantly decreased the metabolic viability of Jurkat compared to the cell lines derived from solid tumor, leading to the inference that the cytotoxic effect of compound **1** is significantly higher against cell lines derived from liquid tumor than those from solid tumor. However, these differences were not consistently observed for the other concentrations tested.

Immunotherapy is one of the expanding fields in cancer treatment [7,9]. The effect of xanthone **1** on macrophage functions was tested for unstimulated, LPS-stimulated, and IL-4-stimulated macrophages in order to achieve the M0, M1, and M2 phenotypes, respectively. Supernatants of xanthone **1**-treated macrophages were tested against cancer cell lines, but they did not affect the metabolic viability of the cancer cells when macrophages were not stimulated or were stimulated with LPS. However, when macrophages were stimulated with IL-4, an increase in cytotoxicity against cancer cell lines was observed for supernatants of compound **1**-treated macrophages (*p* < 0.05) compared to the control. Therefore, the interference of xanthone **1** on cytokine production by human macrophages characteristic of M1 profile (IL-1β and TNF-α) or M2 profile (IL-10 and TGF-β) [7,9,10] was evaluated.

The effects of TGF-β are mainly dependent on the cell type and cell microenvironment [21], and TGF-β1 inhibits the expression of proinflammatory cytokines when macrophages are challenged by LPS [22]. Xanthone **1** treatment caused no significant difference in TGF-β1 expression by THP-1 macrophages, irrespective of the stimulation. IL-10 is an anti-inflammatory cytokine, but its effect has been described to change in accordance with the target cell [21]. IL-10 production was not significantly affected on unstimulated macrophages treated with xanthone **1**, and the same was observed when macrophages were stimulated by LPS or IL-4. NO is produced by macrophages and several cancer cells [23]. However, its functions in cancer are controversial as NO is able to act as a pro- or antitumor molecule depending on its concentration [23,24]. Regarding NO, xanthone **1** caused only a slight decrease in its production by LPS-stimulated RAW264.7 murine macrophages. Xanthone **1** had no relevant effect on RAW 264.7 macrophage viability, leading to the conclusion that the decrease in NO production was not a consequence of cell death.

The studies on the effect of xanthone **1** on cytokine expression and NO production were not able to explain the dependent increase of the antitumor effect of IL-4-stimulated macrophage supernatants against the cancer cell lines. Recent studies have demonstrated that Tap-73 has a role in macrophage polarization, innate immunity, and inflammatory response [17]. In fact, in a very recent study, Wolfsberger and coworkers reported that Tap73 regulates macrophage accumulation and phenotype in breast cancer through inhibition of the NF-κB pathway [25]. To the best of our knowledge, this is the first study reporting the synergistic effect of IL-4 plus xanthone **1** in macrophage antitumor activity, and additional studies are needed in order to understand the molecular mechanism behind it. Additional studies should also include more solid cancer cell lines in order to see if the synergistic effect of IL-4 plus xanthone **1** in macrophage antitumor activity is similar to what we have observed for prostate and cervical cancer cell lines.

## 4. Materials and Methods

### 4.1. Reagents

The reagents and media were acquired as follows: Roswell Park Memorial Institute-1640 (RPMI-1640) medium with Ultraglutamine from Lonza (Verviers, Belgium); fetal bovine serum (FBS) from GE Health Care Life Sciences (Logan, UT, USA); 2-mercaptoethanol from VWR International (Leuven, Belgium); Dulbecco’s modified Eagle medium/F-12 nutrient mixture (Ham) (DMEM/F-12; 1:1) from Gibco (Paisley, UK); phosphate-buffered saline (PBS) from Fisher Reagent (Geel, Belgium); dimethyl sulfoxide (DMSO) and phosphoric acid from Merk (Darmstadt, Germany); dimethylformamide (DMF) from Romil (Cambridge, UK); uncoated ELISA kits from Invitrogen by Thermo Fisher Scientific (Vienna, Austria); TripleXtractor and RNA Kit (Blood and Cultured Cells) from GRiSP (Porto, Portugal); recombinant human IL-4 from R&D Systems (Minneapolis, MN, USA); and High Capacity RNA-to-cDNA Kit and Master Mix form Applied Biosystems (Foster City, CA, USA). When not specified, the reagents were from Sigma-Aldrich (St. Louis, MO, USA).

### 4.2. 1-Carbaldehyde-3,4-dimethoxyxanthone (***1***)

1-Carbaldehyde-3,4-dimethoxyxanthone (**1**) was synthetized by our group at the Laboratory of Organic and Pharmaceutical Chemistry, Department of Chemical Sciences, Faculty of Pharmacy, University of Porto, as previously described [26]. The powered compound was dissolved in methyl sulfoxide (Acros Organics), stored at −20 °C, and diluted before each assay at the desired concentration in the appropriate culture media.

### 4.3. Cell Lines and Cell Culture

PC-3 cell lines were obtained from the European Collection of Cell Cultures (ECCAC) and LNCaP from the American Type Cell Culture (ATCC). The other cell lines were kindly provided by Maria José Oliveira (THP-1 and HeLa), Tumor and Microenvironment Interactions Group, Institute for Investigation and Innovation in Health, i3S, Porto, Portugal; Henrique Almeida (Jurkat), Ageing and Stress group, Institute for Investigation and Innovation in Health, i3S, Porto, Portugal; and Maria São José Nascimento (RAW 264.7), Laboratory of Microbiology, Biological Sciences Department, Faculty of Pharmacy, University of Porto, Portugal. The complete culture medium for cancer cells lines was composed of RPMI-1640 supplemented with 10% of FBS and 1 μg/mL gentamicin. For the THP-1 cell line, the complete culture media described was supplemented with 2-mercaptoethanol (0.05 mM). The RAW 264.7 cell line was cultured in DMEM/F-12 supplemented with FBS and gentamicin as already cited. Cell incubation was performed in a 5% CO_2_ incubator at 37 °C with humidified atmosphere.

### 4.4. Cytotoxic Assay (MTT) for Adherent Cancer Cell Lines

The MTT colorimetric assay was conducted based on the original procedure proposed by Mosmann [27], with a few modifications. The cell lines (HeLa, PC-3, or LNCaP) were seeded at a concentration of 1.5 × 10^4^ cells/well (96-well flat-bottom culture plate). After 24 h incubation to allow cell adherence [28], supernatants were removed and compounds added at the testing concentrations. An untreated control and doxorubicin-treated cells (positive control) [29] were included. Cells were incubated for a further 48 h [28]. Later, supernatants were removed and cells were washed. An MTT solution in culture media was added to the cells (0.2 mg/mL) followed by 4 h incubation [2]. After this time, supernatants were rejected and DMSO was used to solubilize the MTT formazan product [28]. After 10 min incubation with shaking, absorbance was measured using STAT FAX 3200 at 545/630 nm. Cytotoxicity was calculated as the percentage of cellular metabolic viability inhibition using the following formula:

100 − (abs sample/abs control × 100)(1)

### 4.5. Cytotoxic Assay (MTT) for Nonadherent Cancer Cell Lines

Jurkat cells (1.5 × 10^4^ cells/well) were incubated for 24 h [28]. After that period, an equal volume (50 μL) of the desired test concentration of the compounds was added and cells incubated for an additional 48 h period [28]. Untreated cells, doxorubicin-treated cells, a compound blank, and a medium blank (negative control) were also included. After incubation, an MTT solution was added in order to obtain 0.2 mg/mL concentration in each well [2]. After 4 h incubation to allow MTT reduction by viable cells, 50 μL of 20% SDS solution prepared in DMF/H_2_O (1:1) was added to the wells to dissolve formazan [2], and the absorbance was read as previously described. Cytotoxicity was calculated as the percentage of cellular metabolic viability inhibition using the following formula:

100 − [(abs sample-abs blank)/(abs control − abs negative control) × 100](2)

### 4.6. THP-1 Macrophage Phenotype Differentiation and Xanthone ***1*** Conditioned Macrophage Supernatants

THP-1 human leukemic monocyte cell line (1 × 10^6^ cell/mL) was incubated with phorbol 12-myristate 13-acetate (PMA, 0.1 μg/mL) for 72 h in order to promote differentiation of the cells into macrophages [30]. To achieve an M0 phenotype, cells were washed and incubated for another 24 h with culture media [30,31]. The interference of compounds with different macrophage phenotypes was obtained by treating the macrophages with the desired concentration of the compound for 24 h [32] in three different conditions: unstimulated, LPS-stimulated (1 μg/mL) [32], and IL-4-stimulated (20 ng/mL) [30]. Untreated macrophages, stimulated or not, were also used. Macrophage supernatants were removed and used immediately for other tests or frozen at −70 °C until use.

### 4.7. Effect of Conditioned Macrophage Supernatants on Cancer Cell Viability

Macrophage supernatants (100 μL) were used in the cytotoxic assay (MTT) for adherent cancer cell lines as already described.

### 4.8. ELISA Assay for Quantification of Cytokine Expression

The quantification of cytokines (IL-1β, TNF-α, IL-10, and TGF-β1) was performed on conditioned macrophage supernatants using uncoated ELISA kits following the manufacturers’ instructions.

### 4.9. Assay for Quantification of Cytokine mRNA Expression

To quantify mRNA cytokine expression, THP-1 macrophages (M0) were obtained as described in Section 4.6. The interference of compounds with cytokine mRNA expression was obtained by treating the macrophages with the desired concentration of the compound for 6 h [30] in three different conditions: unstimulated, LPS-stimulated, and IL-4-stimulated. Untreated macrophages, stimulated or not, were also used. After incubation, supernatants were aspirated and cells were washed. The mRNA was isolated using TripleXtractor reagent, and samples were stored at −70 °C until use. After separation of the RNA fraction, the samples were purified using the GRS Total RNA Kit (Blood and Cultured Cells). RNA concentration and purity were assessed by the NanoDrop Lite spectrophotometer (Thermo Scientific^®^, Waltham, MA, USA). mRNA samples were the template for cDNA synthesis, using the High Capacity RNA-to-cDNA Kit. The reactions were performed in the StepOneTM qPCR Real-Time PCR instrument containing 1 × Master Mix, 1 × probes (TaqMan^®^ Gene Expression assays IL-1β, Hs01555410_m1; TNF-α, Hs02621508_s1; IL-10, Hs00961622_m1; and TGF-β1, Hs00998133_m1—all from Applied Biosystems, Foster City, CA, USA). The housekeeping gene B2M (TaqMan^®^ Hs99999907_m1; Applied Biosystems, Foster City, CA, USA) served as endogenous reference, expressed at a consistent level to normalize the results. The data analysis was carried out using the StepOneTM Software v2.2 (Applied Biosystems, Foster City, CA, USA) with the same baseline and threshold set for each plate to generate quantification cycle values (Cqs) for all the mRNAs in each sample.

### 4.10. Nitric Oxide Production Assay

RAW 264.7 cell line concentration was adjusted to 1 × 10^6^ cell/mL, and 200 µL was added to a 96-well culture plate. Cells were incubated for 2 h to enable cellular adhesion [1]. Supernatants were rejected, and equal volumes of LPS solution (final well concentration of 1.5 µg/mL) and compound test dilutions were added simultaneously (except when other conditions are explicitly stated). Cells were then incubated for a total of 24 h, and the supernatants (100 µL) were transferred to a new 96-well flat-bottom plate. Griess Reagent (1:1 solution of 1% *w*/*v* sulfanilamide solution in phosphoric acid (5% *v*/*v*) and naphtylethylenediamide (0.1%) in deionized water) was prepared and added (100 µL) to all the wells, followed by 10 min incubation at room temperature in the dark [1]. The optical density was measured at 545/630 nm (STAT FAX 3200), and quantification of the inhibition of nitrite production was calculated by the following formula:

Inhibition of NO production (%) = 100 − [(abs sample-abs blank)/(abs control − abs negative control) × 100](3)

### 4.11. Statistical Analysis

For statistical analysis, IBM SPSS Statistics 26.0 for Windows was used. Data is presented as the mean ± SEM. Media ± 2 SD was used to get meaningful results in order to perform statistical evaluation of the cytotoxicity activity of xanthone **1** against cancer cell lines. Normality of data distribution was validated using the Shapiro–Wilk test of normality, and homogeneity of variance assumption was confirmed using Levene’s test. One-way ANOVA was performed for the experimental cytotoxicity analysis with Bonferroni’s correction post hoc test. The Mann–Whitney test was used for analysis of statistical differences in cytokine expression. Statistical significance was considered for *p* < 0.05.

## 5. Conclusions

Xanthone **1** is a potent antitumor compound against prostate and cervical cancer cell lines. Xanthone **1** also has a strong antitumor effect against the Jurkat cell line.

Besides the direct effect against cancer cells, xanthone **1** interferes with macrophage functions and increases its effectiveness against the metabolic viability of the cancer cell lines when stimulated with IL-4. However, in this study, it was not possible to elucidate the mechanism by which xanthone **1** modulates the cytotoxicity of human macrophages against cancer cells after macrophage induction of an M2 pro-tumoral phenotype.

More studies are needed to elucidate the anticancer cytotoxic and immunomodulatory effect of xanthone **1**.

## Figures and Tables

**Table 1:** Effect of 1-carbaldehyde-3,4-dimethoxyxanthone (**1**) on the metabolic viability of cancer cell lines.

Concentration (μM)	Decrease of Metabolic Viability (% of Control)			
	HeLa	LNCaP	PC-3	Jurkat
20	31.2 ± 8.3	60.6 ± 5.9	72.0 ± 4.0	99.1 ± 7.8
10	26.5 ± 8.6	51.8 ± 6.0	65.5 ± 5.0	T.I.
5	10.2 ± 1.3	35.9 ± 10.4	68.6 ± 1.5	78.2 ± 10.4

Values represent mean ± SEM (*n* = 3–5). T.I., total inhibition. Doxorubicin (5 µM) was used as control with a cytotoxicity (% of control) of 82.8 ± 2.5%, 62.5 ± 1.8%, 73.2 ± 2.8%, and 111.4 ± 2.8% for HeLa, LNCaP, PC-3, and Jurkat, respectively.

**Table 2:** Effect of 1-carbaldehyde-3,4-dimethoxyxanthone (**1**) on nitric oxide (NO) production and viability of LPS-stimulated RAW264.7 macrophages.

Concentration (μM)	NO Production Inhibition (% of Control)	RAW 264.7 Viability (% of Control)
10	28.1 ± 3.7	98.4 ± 4.6
5	15.3 ± 3.0	N.I.

Values represent mean ± SEM (*n* = 3–5). Dexamethasone (5 μM) was used as positive control for NO production inhibition (58.1 ± 7.8%). NO, nitric oxide; LPS, lipopolysaccharide; N.I., no inhibition.
